# Melatonin and KNO_3_ Application Improves Growth, Physiological and Biochemical Characteristics of Maize Seedlings under Waterlogging Stress Conditions

**DOI:** 10.3390/biology11010099

**Published:** 2022-01-09

**Authors:** Shakeel Ahmad, Guo-Yun Wang, Ihsan Muhammad, Muhammad Zeeshan, Xun-Bo Zhou

**Affiliations:** Guangxi Colleges and Universities Key Laboratory of Crop Cultivation and Tillage, National Demonstration Center for Experimental Plant Science Education, Agricultural College of Guangxi University, Nanning 530004, China; shakeel1287@hotmail.com (S.A.); yuanyiwanggy@yeah.net (G.-Y.W.); ihsanagrarian@yahoo.com (I.M.); 11616102@zju.edu.cn (M.Z.)

**Keywords:** melatonin, KNO_3_, plant growth, enzymes, photosynthesis, waterlogging stress, maize

## Abstract

**Simple Summary:**

Waterlogging is one of the serious abiotic stresses that retards plant growth and reduces crop productivity. Therefore, exploring effective ways for alleviating the impacts of waterlogging stress has important theoretical and practical applications. Melatonin is a pleiotropic molecule that influences many diverse actions to enhance plant growth. Therefore, investigating efficient waterlogging mitigation measures has both theoretical and practical significance. The objectives of the present research were to examine the efficiency of melatonin and KNO_3_ seed soaking and foliar application on alleviating the waterlogging inhibited growth performance of maize seedlings. The results showed that melatonin and KNO_3_ significantly improved the plant growth and biochemical parameters of maize seedlings under waterlogging stress conditions. Overall, the application of 100 µM melatonin with 0.50 g KNO_3_ via seed soaking and foliar spray can be used as a potential mitigating strategy for improving the growth of maize seedlings and alleviating the ill effects of waterlogging stress.

**Abstract:**

Waterlogging is one of the serious abiotic stresses that inhibits crop growth and reduces productivity. Therefore, investigating efficient waterlogging mitigation measures has both theoretical and practical significance. The objectives of the present research were to examine the efficiency of melatonin and KNO_3_ seed soaking and foliar application on alleviating the waterlogging inhibited growth performance of maize seedlings. In this study, 100 µM melatonin and different levels (0.25, 0.50 and 0.75 g) of potassium nitrate (KNO_3_) were used in seed soaking and foliar applications. For foliar application, treatments were applied at the 7th leaf stage one week after the imposition of waterlogging stress. The results showed that melatonin with KNO_3_ significantly improved the plant growth and biochemical parameters of maize seedlings under waterlogging stress conditions. However, the application of melatonin with KNO_3_ treatments increased plant growth characteristics, chlorophyll content, and the net photosynthetic rate at a variable rate under waterlogging stress. Furthermore, melatonin with KNO_3_ treatments significantly reduced the accumulation of hydrogen peroxide (H_2_O_2_) and malondialdehyde (MDA), and it decreased the activity of pyruvate decarboxylase and alcohol dehydrogenase, while increasing enzymatic activities and soluble protein content of maize seedlings under waterlogging stress conditions. Overall, our results indicated that seed soaking with 100 µM melatonin and 0.50 g KNO_3_ was the most effective treatment that significantly improved the plant growth characteristics, chlorophyll content, photosynthetic rate, and enzymatic activity of maize seedling under waterlogging conditions.

## 1. Introduction

Waterlogging is a major agricultural constraint that limits plant growth, reduces agricultural crop yield, and is exacerbated by global climate change [1]. Excessive waterlogging damages roots, reduces water intake, eventually causes chlorosis, and wilts the plants [2]. It was estimated that waterlogging stress resulted in nearly 40–80% of the crop yield loss in the area greater than 17 million km^2^ [3]. Waterlogging has become a prevalent phenomenon, particularly in southern China [4]. Excessive water causes saturated soil and an inadequate oxygen supply, leading to hypoxic conditions in plant root systems. Plants produce reactive oxygen species (ROS) in response to hypoxia, causing several physiological, phenotypic and metabolic disturbances, especially a fermentative metabolism [5]. The induction of a fermentative metabolism is regarded as an adaptive response to maintain energy synthesis. Alcohol dehydrogenase and pyruvate decarboxylase are the two key enzymes in this process. However, the main end product of the fermentative metabolism is ethanol, which is harmful to the plant root system [6]. With the global production of 1099.61 million metric tons, maize is very susceptible to waterlogging stress during its vegetative stage, resulting in a considerable yield reduction [1,7]. Waterlogging predominantly affects plants by triggering rhizosphere degeneration, which leads to a decline in root activity and a reduction in water and nutrient absorption, particularly ion absorption [8]. Plants under waterlogging stress may have reduced uptake of nitrogen, an essential element for the synthesis of amino acids, proteins, enzymes, and nucleic acids [9]. Following waterlogging, plants often exhibit N deficiency, so that leaves lower on shoots show signs of wilting and yellowing, ultimately inhibiting plant growth and development [10]. Waterlogging also affects nitrogen metabolism enzymes and results in decreased chlorophyll content, consequently reducing the photosynthetic capacity of plants [11]. Furthermore, leaf stomatal closure restricts gas exchange and increases ROS accumulation under waterlogging stress conditions [2]. Excessive ROS can cause degradation of chloroplast plasma membranes, destroying photosynthetic systems, thereby constraining plant growth and development [11,12]. Superoxide dismutase (SOD), catalase (CAT), peroxidase (POD), and ascorbate peroxidase (APX) are the most common antioxidant enzymes that collaborate to defend against oxidative stress [13]. However, the most important adaptive mechanism is a well-balanced antioxidant defense system consisting of both enzymatic and non-enzymatic enzymes to facilitate the scavenging of the ROS [14,15].

Nitrogen (N) is a vital source and crucial nutrient for plant growth and development because it affects the composition of proteins, nucleic acids, hormones, and other essential compounds [16]. In the last 50 years, the application of synthetic N fertilizer has increased dramatically to meet the agricultural demands of the growing population [17]. Nitrogen fertilizer application rates have recently increased significantly in Chinese intensive agricultural systems, mostly for crop production. Higher N fertilizer input increased crop yield, but it also reduced plant N usage efficiency, and excess N can have major environmental and human health repercussions [18]. As a result, maintaining crop growth and production while lowering nitrogen fertilizer ratios poses a significant challenge to agriculture for long-term survival [19]. Waterlogging also constrains soil quality, and muddy soils prevent the proper distribution of ground fertilizer application. Foliar and seed priming nitrogen application is a quick, simple, and effective method of nitrogen fertilizer replenishment. Regarding in-ground fertilizers, urea is usually the source for nitrogen, and appropriate levels of sprayed N in the form of KNO_3_ on leaves may temporarily alleviate N deficiency in plants under abiotic stress [4,16]. Research indicates that the foliar application of N significantly improves antioxidant activity (i.e., reducing ROS) and effectively alleviates waterlogging stress in canola plants [20].

Melatonin (*N-acetyl-5-methoxytryptamine*) is a plant hormone that regulates a wide range of biological and biochemical metabolic activities under abiotic stress conditions [21]. Previous research indicated that (*MzASMT*) overexpression boosted melatonin production and improved drought tolerance in transgenic *Arabidopsis thaliana* plants [22]. Melatonin functions as a growth regulator and an antioxidant during the diverse developmental process under abiotic stress conditions [23]. Melatonin works as an anti-stress agent against abiotic stresses such as extreme temperatures, drought, salinity and hypoxia [21]. Moreover, exogenous melatonin enhances the activity of antioxidant enzymes while decreasing H_2_O_2_ and MDA content to enhance stress tolerance in plants [24]. Exogenous melatonin has been shown to improve tolerance to waterlogging stress by maintaining aerobic respiration, retaining photosynthetic capacities, and minimizing oxidative damage to plants [2,25]. Waterlogging stress makes it more difficult for plants to absorb nutrients, although melatonin can help by boosting nutrient absorption and metabolism [2]. Melatonin is considered an effective molecule to protect plants against stress.

However, the mechanisms of melatonin on plant N uptake and metabolism under waterlogging stress conditions remain unclear. Therefore, additional research is needed to explore the potential positive effects of melatonin on crop development and the mechanism of mitigating the detrimental effects of waterlogging stress. Thus, the present study was conducted to examine the effect of melatonin in combination with different levels of potassium nitrate (KNO_3_) as seed soaking and foliar application on the growth and biochemical characteristics of maize seedlings subjected to waterlogging stress conditions.

## 2. Materials and Methods

### 2.1. Plant Material, Experimental Design, and Location

This research was carried out in a glass shed under natural light conditions in the experimental station of Guangxi University, Nanning, China, in June 2021. Maize (*Zea mays*) seeds variety Wanchuan-1306 was used in the experiment. Before sowing, healthy maize seeds were first disinfected with sodium hypochlorite solution for 15 min and then thoroughly washed several times with distilled water. The pots were arranged in a randomized complete block design, healthy maize seeds were selected and five seeds were planted in each pot. The plastic pots (23.6 cm diameter, 24.5 cm height) were filled with 8 kg of arable topsoil. The physical-chemical properties of the collected soil are shown in Table 1. The optimum level of melatonin (100 µM) was selected based on our previous experiment. Two application methods were used: seed soaking (F) and foliar spray (S). The treatments were: Control 1 not waterlogging (NWL), Control 2 waterlogging (WL), Control 3 WL + 100 µM Melatonin (WLM), WLM + 0.25 g KNO_3_ (WLMN1), WLM + 0.50 g KNO_3_ (WLMN2), and WLM + 0.75 g KNO_3_ (WLMN3). The waterlogging stress was imposed at the 5th leaf stage of maize seedling for one week and afterward, at the 7th leaf stage, foliar treatment was applied. Each group of treatments contained three replications. After one week of treatment, the samples were collected from each treatment for different tests, immediately processed in liquid nitrogen and stored at −80 ℃ for further analysis.

### 2.2. Plant Sampling and Measurements

#### 2.2.1. Growth Parameters

After one week of treatment and the application of waterlogging stress, the plants were collected from each treatment. A tape meter was used to measure the plant height and leaf area per plant (cm^2^) to detect growth changes. Root length was measured by root image analysis using WinRHIZO 2003a software (Regent Instruments, Québec, QC, Canada). The sampled plants were separated into shoots, stem and roots, and weighed after drying at 75 °C for 72 h. The root-to-shoot ratio was calculated by using the following formula:

$$Root\;to\;shoot\;ratio=\frac{Root\;dry\;weight}{Shoot\;dry\;weight}$$

#### 2.2.2. Photosynthetic Rate and Chlorophyll Content

The net photosynthetic rate was measured using a portable infrared gas analyzer photosynthetic system LI-6800XT (LI-COR, Biosciences, Lincoln, NE, USA), on a sunny day between 10:00 and 12:00 p.m. The method of Ahmad et al. [26] was used to determine the chlorophyll pigments. The middle portion of fresh ear leaves (0.1 g) was cut into small pieces then put in tubes, and 10 mL of a 4.5:4.5:1 solution of ethanol, acetone, and distilled water was added and kept in the dark for 24 h. The absorbance of the sample was measured at 663 nm and 646 nm using a UV-spectrophotometer and expressed as mg g^−1^ fresh weight.

#### 2.2.3. Assay of Antioxidant Activity

For the determination of antioxidant enzyme activities, 0.2 g of fresh leaf sample was homogenized with 5 mL phosphate buffer (0.1 M, pH 6.8). The mixed liquid sample was centrifuged at 12,000× *g* for 20 min at 4 °C. The resultant supernatants were used for SOD, POD, CAT, and APX activities, and the results were presented as U mg^−1^ min^−1^ FW.

The antioxidant enzyme activity of SOD was detected according to the method of Shi et al. [27]. POD activity was measured by the changes in absorbance at 470 nm due to guaiacol oxidation following the protocol of Ma et al. [28]. CAT was measured at 240 nm according to Bilal et al. [29]. The APX activity was measured using the method of Hossain et al. [30] and the absorbance of the reaction mixture was read immediately at 290 nm with a UV spectrophotometer.

#### 2.2.4. Determination of H_2_O_2_, MDA Content

The measurement of H_2_O_2_ production was performed by extracting H_2_O_2_ from leaves according to procedure of Habiba et al. [31]. A total of 0.1 g of leaf sample was pulverized and extracted with 5 mL of 0.1 percent TCA, then centrifuged for 15 min at 12,000× *g*. After that, 1 mL of 1 M potassium iodide and 0.5 mL of 10 mM phosphate buffer (pH 7.0) were added to 0.5 mL of supernatant and the absorbance was measured at 390 nm with a UV spectrophotometer. Malondialdehyde content was measured by following the method of Biswojit et al. [32]. MDA content was then determined by spectrophotometer (Evolution 300, Thermo Electron Scientific Instruments LLC, Madison, WI, USA) at 532 nm, and the nonspecific turbidity was calculated by subtracting the absorbance at 600 nm.

#### 2.2.5. Soluble Protein and Soluble Sugar Contents Measurement

The amount of soluble protein was determined following the Coomassie brilliant blue G-250 staining method, as explained by Chen et al. [33] and expressed as mg g^−1^ fresh weight. The soluble sugar content was determined using the anthrone method [34]. Briefly, endogenous soluble total sugars in plant leaves were extracted using an anthrone reagent, and the absorbance was measured at 630 nm.

#### 2.2.6. Determination of Nitrogen Metabolism Enzyme Activities

Glutamine Synthetase (GS) and Glutamate Synthase GOGAT activity were measured according to the method of Wang et al. [35], using GS and GOGAT test kits (BC0915 and BC0070) purchased from the Beijing Solarbio Science and Technology Co., Ltd., Beijing, China. The Nitrate Reductase (NR) and Glutamate Dehydrogenase (GDH) activity were measured the method of Sahay et al. [36], with some modifications by using the NR and GDH test kits (BC0080 and BC1460), purchased from Beijing Solarbio Life Science and Technology Co., Ltd., Beijing, China.

#### 2.2.7. Determination of the ADH and PDC Enzyme Activities

The method of Zanandrea et al. [37] and Gimeno et al. [38] was used to determine the activity of pyruvate decarboxylase (PDC) using a 0.05 M Tris-HCl buffer (pH 7.5) containing 1 mM dithiothreitol and 2% PVP (*w*/*v*). Alcohol dehydrogenase (ADH) activity was measured by the method of Goyal et al. [1], using a 100 mM HEPES buffer containing 2 mM dithiothreitol (pH 6.5).

#### 2.2.8. Statistical Analysis

The experimental data were sorted and calculated using Excel 2019 (Microsoft Corp., Redmond, WA, USA), and were subjected to an analysis of variance (ANOVA) using SPSS 20 Statistics (SPSS Inc., Chicago, IL, USA). Comparisons between means were carried out using Least Significance Difference (LSD) test at a *p* ≤ 0.05. Graph Pad prism 7.00 was used to illustrate the figures.

## 3. Results

### 3.1. Effect of Nitrate on Growth Characteristics of Maize Seedlings Treated with Melatonin under Waterlogging Stress Conditions

Waterlogging (WL) stress reduced the dry biomass accumulation and root length of maize seedlings compared to control (NWL and WLM). The application of melatonin with KNO_3_ significantly enhanced the dry biomass accumulation and root length of maize seedlings under waterlogging stress conditions, as compared to WLM (Table 2). Our results revealed that waterlogging stress reduced the dry biomass of root by 44.08% and 46.76%, stem by 36.21% and 56.17%, leaf by 47.01% and 44.71%, root to shoot ratio by 42.26% and 50.42% and root length by 28.05% and 22.15% in both the foliar and seed soaking methods as compared to NWL. The application of melatonin with KNO_3_ as a foliar spray and seed soaking facilitated the dry biomass of root, stem, leaves, and root length of maize seedlings, which were significantly greater than that in respective waterlogging stress controls (WL and WLM). Melatonin with KNO_3_ treatment (WLMN2) as a foliar spray and seed soaking increased the dry biomass of root by 51.74% and 70.21%, stem by 55.35% and 52.95%, and leaves by 65.29% and 64.68%, respectively, compared to WL (Table 2). The root length showed a sustainable expansion with the application of melatonin with KNO_3_ as a foliar spray. The highest root length was observed in treatment WLMN2 but it was statistically similar to WLMN3 when compared with WL.The greater root length was achieved by seed soaking treatment WLMN2 by 17.83% compared with WL (Table 2). From the overall growth characteristics results, we found that melatonin with KNO_3_ as a seed soaking showed higher biomass accumulation and root length than foliar treatments under waterlogging stress conditions.

### 3.2. Effect of Nitrate on Plant Height and Leaf Area per Plant of Maize Seedlings Treated with Melatonin under Waterlogging Stress Conditions

**Figure 1:**
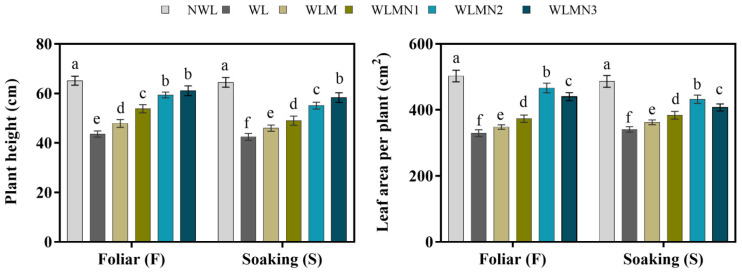
Effect of melatonin with KNO_3_ on plant height and leaf area per plant (cm^2^) of maize seedlings under waterlogging stress conditions. Data are presented as mean ± SD (*n* = 3, biological replicates). Different small letters (a–e) in each column indicate significant differences at *p* ≤ 0.05 (least significant difference (LSD) test).The abbreviations of treatment names are the same as those described in Table 2.

Waterlogging strongly reduces the plant height, this inhibition was concomitantly counteracted by increasing levels of KNO_3_. Our results showed that the melatonin with KNO_3_-treated maize seedlings attained taller plant heights and larger leaf areas than WL and WLM. In foliar application, treatment WLMN3 increased plant height by 40.06% and treatment WLMN2 enlarged the leaf area per plant by 41.54% compared with WL (Figure 1). However, in the seed soaking method, the tallest plant height was achieved by WLMN3 treatment (37.28%) and largest leaf area per plant of 26.69% was observed in treatment WLMN2 as compared with WL (Figure 1).

### 3.3. Effect of Nitrate on Photosynthetic Rate and Chlorophyll Content of Mazie Seedlings Treated with Melatonin under Waterlogging Stress Conditions

**Figure 2:**
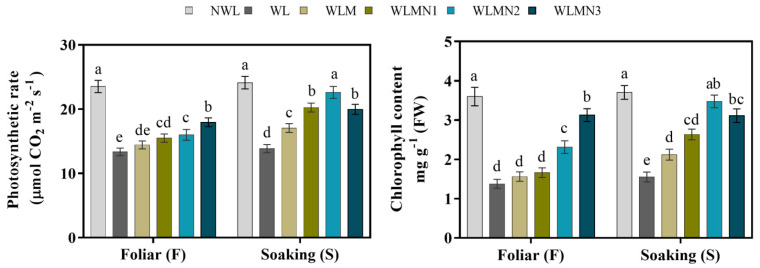
The photosynthetic rate and chlorophyll content of maize seedlings under waterlogging stress conditions. Data are presented as mean ± SD (*n* = 3, biological replicates). Different small letters (a–e) in each column indicate significant differences at *p* ≤ 0.05 (least significant difference (LSD) test). The abbreviations of treatment names are the same as those described in Table 2.

Waterlogging (WL) significantly decreased the photosynthetic rate and chlorophyll contents compared with controls (NWL and WLM). Our results showed that the application of melatonin with KNO_3_ significantly increased the photosynthetic rate and chlorophyll content of maize seedlings compared with WLM. Among foliar treatments, WLMN3 showed the most promising effect, which increased the photosynthetic rate and chlorophyll content by 34.51% and 126.95% compared with WL (Figure 2). However, among seed soaking treatments, WLMN2 showed the highest photosynthetic rate (63.12%) followed by WLMN3, compared to WL. The maximum chlorophyll content of 124.13% was attained by seed soaking treatment WLMN2, and was statistically similar to WLMN3 and NWL (Figure 2).

### 3.4. Effect of Nitrate of on Antioxidant Enzymes Activities of Maize Seedlings Treated with Melatonin under Waterlogging Stress Conditions

**Figure 3:**
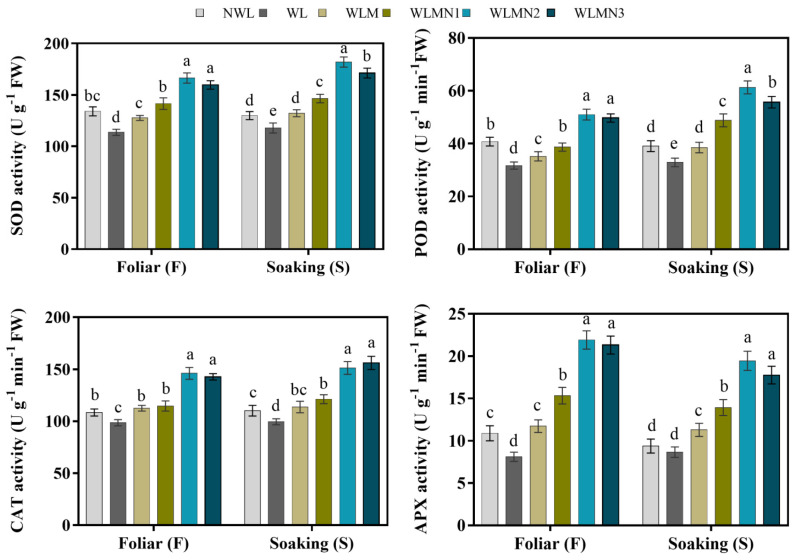
Antioxidant enzyme (SOD, POD, CAT and APX) activities of maize seedlings under waterlogging stress conditions. Data are presented as mean ± SD (n = 3, biological replicates). Different small letters (a–e) in each column indicate significant differences at *p* ≤ 0.05 (least significant difference (LSD) test).The abbreviations of treatment names are the same as those described in Table 2.

Waterlogging stress (WL) markedly reduced the antioxidant enzyme activity compared to NWL and WLM. However, melatonin with KNO_3_ significantly improved antioxidant enzyme activity in maize seedlings subjected to waterlogging stress compared to NWL and WLM (Figure 3). The application of melatonin with KNO_3_ treatment showed the same pattern for improving the various antioxidant enzyme activities. However, compared with WL and the foliar spray of melatonin with KNO_3_ treatment, WLMN2 significantly increased the SOD, POD, CAT and APX activities by 46.42%, 60.99%, 48.14% and 170.09% respectively, followed by WLMN3. Seed soaking treatment WLMN2 enhanced the antioxidant enzymes activity of the SOD by 56.87%, POD by 86.33%, CAT by 51.94% and APX by 124.51%, compared with WL. The overall results demonstrated that foliar treatments WLMN2 and WLMN3 were statistically similar in all antioxidant enzymes. In the seed soaking treatments, WLMN2 and WLMN3 showed statistically similar results for the CAT and APX activities of maize seedlings under waterlogging stress conditions (Figure 3).

### 3.5. Effect of Nitrate on H_2_O_2_, MDA, Soluble Protein and Soluble Sugar Content of Maize Seedlings Treated with Melatonin under Waterlogging Stress Conditions

**Figure 4:**
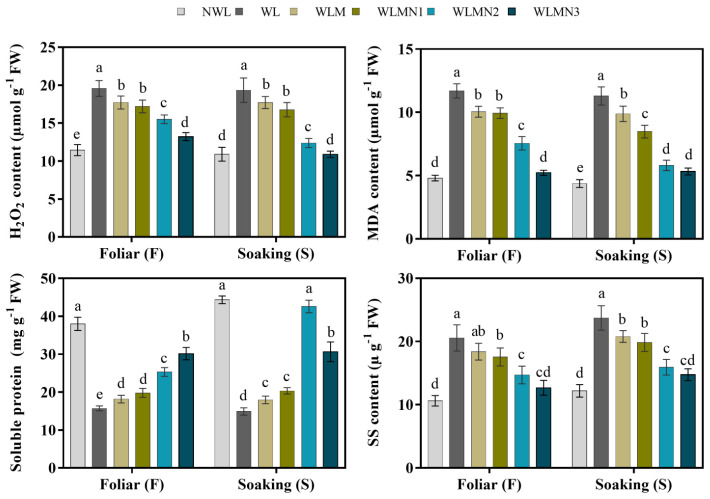
H_2_O_2_, MDA, soluble and soluble sugar content of maize seedlings under waterlogging stress conditions. Data are presented as mean ± SD (*n* = 3, biological replicates). Different small letters (a–e) in each column indicate significant differences at *p* ≤ 0.05 (least significant difference (LSD) test). The abbreviations of treatment names are the same as those described in Table 2.

Waterlogging stress elevated the accumulation of H_2_O_2_ and MDA content in maize seedlings compared with the control (NWL and WLM). Our results showed that melatonin with KNO_3_ significantly reduced the accumulation of H_2_O_2_ and MDA content under waterlogging stress conditions compared to WLM (Figure 4). WL increased H_2_O_2_ and MDA content by 71.05% and 143.51% in foliar spray, while the increases were 77.40% and 158.26% with the seed soaking method. The results also showed that the H_2_O_2_ content of the treatments WLMN1, WLMN2, and WLMN3 were 12.16%, 20.79%, and 32.49% lower in foliar spray, respectively, while in the seed soaking method, the WLMN1, WLMN2, and WLMN3 were 13.32%, 35.96%, and 43.70% compared with WL, respectively (Figure 4).

Waterlogging (WL) significantly decreased the soluble protein content of maize seedlings compared with NWL, WLM and treated plants (Figure 4). Our results showed that melatonin with KNO_3_ increased the soluble protein content at various degrees than WLM. The results demonstrated that soluble protein was significantly increased in treatments WLMN1, WLMN2 and WLMN3 by 26.01%, 61.16%, and 91.90% under the foliar spray, while in seed soaking, the maximum soluble protein content obtained was in the WLMN2 treatment, increasing it by 185.99% compared with WL (Figure 4).

The soluble sugar content of maize seedlings significantly increased in WL compared with NWL. Our results revealed that melatonin with KNO_3_ as a foliar spray and seed soaking treatment significantly reduced the soluble sugar content compared with WLM. As shown in (Figure 4), the foliar treatments WLMN1, WLMN2, and WLMN3 decreased the soluble sugar by 14.54%, 28.41%, and 38.35%, respectively, while seed soaking treatments WLMN1, WLMN2, and WLMN3 reduced the soluble sugar contents by 16.39%, 32.90%, and 37.92%, respectively, compared with WL.

### 3.6. Effect of Nitrate on Nitrogen Metabolism Enzyme Activities of Maize Seedlings Treated with Melatonin under Waterlogging Stress Conditions

**Figure 5:**
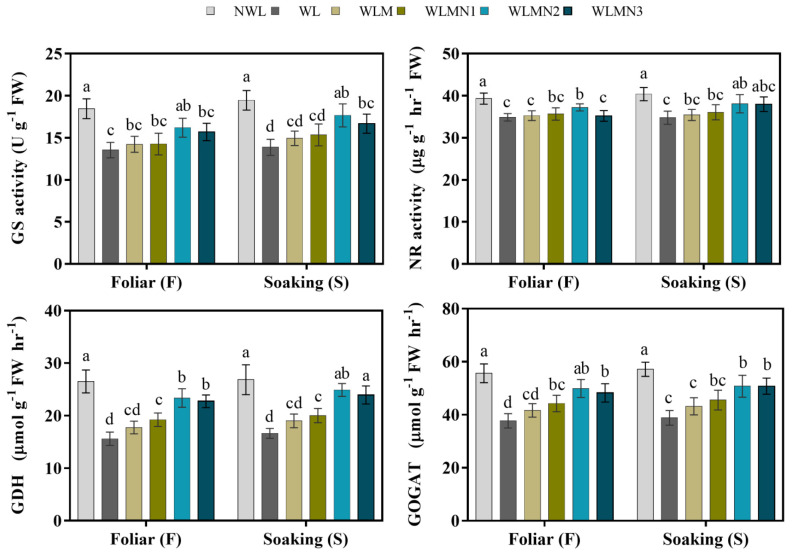
Nitrogen metabolism enzyme activities of maize seedlings under waterlogging stress conditions. Data are presented as mean ± SD (*n* = 3, biological replicates). Different small letters (a–e) in each column indicate significant differences at *p* ≤ 0.05 (least significant difference (LSD) test).The abbreviations of treatment names are the same as those described in Table 2.

Nitrogen metabolism enzymes (GS, NR, GOGAT, and GDH) activities reduced by 26.67%, 11.28%, 32.22%, and 41.14% under WL compared with NWL. Our results showed that melatonin with KNO_3_ increased the nitrogen metabolism activity than WL and WLM (Figure 5). In the present study, our results showed that foliar application of melatonin with KNO_3_ treatment WLMN2 increased the GS by 19.67%, NR by 6.74%, GOGAT by 32.28% and GDH by 49.72% compared with WL. Seed soaking of melatonin with KNO_3_ treatment WLMN2 increased the GS by 27.36%, NR by 9.53%, GOGAT by 30.69%, and GDH activities by 49.63% in maize seedlings, as compared with WL (Figure 5).

### 3.7. Effect of Nitrate on Alcohol Dehydrogenase (ADH) and Pyruvate Decarboxylase (PDC) Activities of Maize Seedlings Treated with Melatonin under Waterlogging Stress Conditions

**Figure 6:**
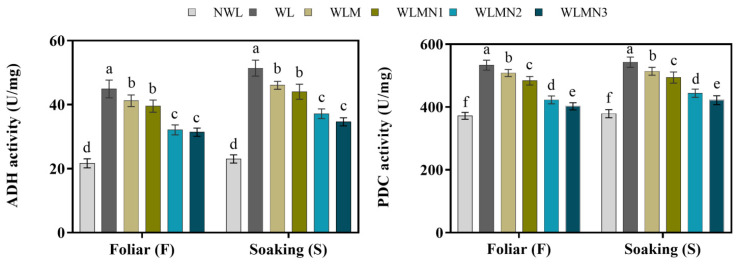
ADH and PDC activities of maize seedlings under waterlogging stress conditions. Data are presented as mean ± SD (*n* = 3, biological replicates). Different small letters (a–e) in each column indicate significant differences at *p* ≤ 0.05 (least significant difference (LSD) test).The abbreviations of treatment names are the same as those described in Table 2.

The ADH and PDC activities of maize seedlings showed the same pattern in both application methods under waterlogging stress conditions. The results showed that WL significantly increased the ADH and PDC activities compared with the NWL, WLM and treated plants (Figure 6). Foliar spray of melatonin with KNO_3_ treatment WLMN2 significantly reduced the ADH activity by 28.46% but was statistically similar to WLMN3, and PDC activity was reduced by 20.77% compared with WL, followed by WLMN3. Seed soaking of melatonin with KNO_3_ treatment WLMN2 significantly decreased the ADH by 27.71% and PDC activity by 18.21% as compared to WL (Figure 6).

## 4. Discussion

Waterlogging is one of the most common environmental issues that restrict plant growth and productivity. As a result of the global warming effect, it is predicted that climate change will result in higher rainfall yearly and thus that waterlogging will be frequently encountered by crops worldwide [2]. This will be a major problem for crops like maize, which are sensitive to waterlogging stress at the early growth stage. Melatonin was reported to enhance plant tolerance against various abiotic stressors including cold, hot, drought, salinity, etc. [13,39]. A previous study also showed that N fertilizer applied in the form of KNO_3_ could alleviate the negative effects of abiotic stress by increasing organic solutes and antioxidant enzyme activities, as well as by reducing the accumulation of ROS [38]. However, no previous studies have been conducted to understand the influence of melatonin with KNO_3_ on waterlogging stress. Therefore, the current study was conducted to evaluate the application of melatonin with KNO_3_ under waterlogging stress conditions. As a result of waterlogging, plants face various morphological and physiological changes, including root and shoot development inhibition, reduced water and nutrient intake, and eventually chlorosis and mortality [40]. The results showed that application of melatonin with KNO_3_ as a seed soaking and foliar spray at the seventh leaf stage during waterlogging stress conditions increases growth, root length, and biomass accumulation of the root, stem, shoot, and leaves of maize seedlings compared with WLM. Waterlogging stress adversely affects the growth of many crops, causing chlorosis and necrosis in leaves, inhibiting shoot and root growth and decreasing dry matter accumulation. However, melatonin with KNO_3_ alleviated waterlogging stress and positively affected plant growth and development [11,41]. The increase in biomass accumulation and growth attributes is endorsed to the improved carbon assimilation due to the enhanced photosynthetic capacity of the treated plants [42]. Our experiment indicated that melatonin with KNO_3_ application effectively enhanced the adaptability of seedlings to waterlogging stress by ameliorating the suppression of growth characteristics. Chlorophyll is an important pigment involved in photosynthesis and plays a key role in absorbing and transmitting light energy. Similarly, carotenoids are pigments that act as a photo protectant and function by releasing the excess energy before it can damage the plants [40]. Waterlogging stress resulted in a decrease to chlorophyll content, which undeniably impairs and reduce photosynthesis, directly affecting plant growth [21]. Melatonin with KNO_3_ serves chlorophyll pigments and as a result improves photosynthesis in the plants grown under abiotic stress conditions [11,41]. Our results showed that WL significantly decreased the chlorophyll content and net photosynthetic rate, as compared to NWL. However, the application of melatonin with KNO_3_ improved the photosynthetic rate and chlorophyll content under waterlogging stress conditions, as compared to WL and WLM. The photosynthesis-preserving and growth-promoting abilities of melatonin have already been demonstrated in various plant species under various abiotic stress conditions [24,43]. The increase in chlorophyll content is partly due to the improved photosynthetic rate of plants [21,44,45]. Various environmental abiotic stresses, such as temperature, salt, water, and heavy metal stress, have been reported to stimulate melatonin accumulation in plant species, and this stimulation has been regarded as a self-defense response to external stimuli via the regulation of leaf senescence, antioxidant systems, and the carbon and nitrogen metabolism in plant species [40,46].

Exogenous application of melatonin could significantly improve photosynthetic performance under waterlogging by reducing ROS accumulation and alleviating membrane lipid peroxidation [47,48]. Previous research reported that nitrogen supplementation could help to maintain enzymatic activities and alleviate the damage to membrane lipids by peroxide under waterlogging stress conditions [11]. In the current study, the MDA content of maize seedlings increased under WL compared with NWL and WLM, suggesting that lipid peroxidation was increased, probably due to ROS accumulation. Melatonin with KNO_3_ improves the tolerance of plants to waterlogging stress, possibly due to antioxidant and ROS scavenging capabilities [49]. Melatonin is a plant growth regulator that regulates the accumulation of other suitable solutes, such as osmolytes, soluble sugars and protein content, in plants under abiotic stress conditions [50]. Our results showed that soluble protein content was significantly reduced under WL treatment as compared to in NWL and WLM. In contrast, the soluble protein content was remarkably increased in the melatonin with KNO_3_-treated plants under waterlogging stress conditions, as compared to WLM. A previous study conducted by Ruiz et al. [51] demonstrated that abiotic stress reduced the soluble protein content by inducing protein hydrolysis and restraining protein synthesis. Melatonin also initiates the biosynthesis of these osmolytes in plants under abiotic stress [52]. The current study showed that the soluble sugar content of maize seedlings significantly increased in WL as compared to the NWL, WLM and melatonin with KNO_3_-treated plants. The osmotic adjustments of waterlogged stressed plants were facilitated by increased osmolyte production, accompanied by an increase in osmolyte concentration. Plants have self-developed strategies to prevent oxidative damage by reducing the osmotic potential of cells by increasing osmotic solutes like soluble sugar [53]. To cope with oxidative damage, plants have evolved the effective defense mechanisms of antioxidant enzyme (SOD, POD, CAT, and APX) activities. Our results revealed that WL markedly reduced the antioxidant enzyme activities compared to NWL and WLM. However, melatonin with KNO_3_ significantly improved the antioxidant enzymes in maize seedlings under waterlogging stress as compared to WLM. Regulation of the antioxidant enzymes is an innate plant response to negate the oxidative stress caused by various external biotic and abiotic stress factors [24,40]. Antioxidant enzyme activity must be maintained at a high level for plants to adapt under waterlogging stress [54]. Our findings agree with previous studies which revealed that waterlogging stress causes oxidative damage in maize seedlings [55,56]. Increasing KNO_3_ levels with the optimum level of melatonin application can also improve antioxidant enzyme (SOD, POD, CAT, and APX) activities in winter rape, cotton, and maize leaves under waterlogging stress conditions [11,57]. Our results demonstrated that the application of melatonin with KNO_3_ could reduce H_2_O_2_ accumulation in maize leaves, minimizing waterlogging-induced oxidative membrane damage.

Exogenous melatonin with KNO_3_ promotes the antioxidant enzymes and enhances the nitrogen metabolism activities of maize under waterlogging stress conditions. The glutathione synthase (GS), nitrate reductase (NR), glutamate synthetase (GOGAT), and glutamate dehydrogenase (GDH) enzymes play important roles in nitrogen metabolism [58]. We found that under WL stress, the GS and NR were reduced compared with NWL and WLM. Application of melatonin with KNO_3_ treatments increased GS and NR activity in the leaves of maize seedlings, as compared with WL and WLM. NR is the main source of nitrogen acquisition in plants and regulates the synthesis of NO (recognized as a centric signaling molecule in plants). In addition, GS also plays an important role in N metabolism. Our results are in agreement with previous studies that reported that melatonin treatment (100 µM) increased the activity of enzymes (NR and GS) related to nitrogen metabolism in maize plants [16,19]. Moreover, the previous study revealed that melatonin treatment improves NR and GS activity in wheat crops as compared to no-treatment applications under abiotic stress conditions [59]. The results of the current experiment also showed that GOGAT and GDH activities significantly declined under waterlogging stress, leading to a significant reduction in nitrogen metabolism enzyme activity and limiting maize seedlings’ normal physiological function. The research of Arora and Bhatla [60] revealed that melatonin directly modulates the activity of NR, GS, GDH, and GOGAT in response to abiotic stress conditions. The increased activities of these enzymes were related to melatonin, which enhanced the antioxidant properties of GS and GOGAT [18]. In this study, we found that melatonin with KNO_3_ enhanced enzyme activity and accelerated the nitrogen metabolism in maize seedlings under waterlogging stress conditions.

ADH and PDC are important components of the ethanol fermentation pathway, and their activity is frequently utilized as a primary indicator of plant waterlogging resistance. Waterlogging-stressed plants can increase the rate of ethanol fermentation by controlling the expression of ADH, PDC, and other associated enzyme genes, which can supply temporary energy for plant growth and development under waterlogging stress conditions [41,61]. The current results indicated that melatonin with KNO_3_ decreased ADH and PDC activity as compared to WL and WLM. Previous research demonstrated that exogenous melatonin reduced the enhanced involvement of key enzymes in alcohol fermentation in peach roots under waterlogging stress conditions [62]. Waterlogging stress damages maize seedlings and changes their metabolism from aerobic to anaerobic respiration. A burst of ROS is generated during this transition, resulting in oxidative damage to the seedlings. However, the application of melatonin with KNO_3_ prevented these changes in plants under waterlogging stress conditions. Another previous study reported that the application of exogenous melatonin modulated anaerobic respiration in apple seedlings under waterlogging stress conditions [13,62]. According to our current findings and the conclusions of previous studies, the mitigating potential of melatonin with KNO_3_ is highly related to the method of application and appropriate level of waterlogging stress conditions.

## 5. Conclusions

In conclusion, waterlogging stress inhibited maize seedling growth and development. However, the application of melatonin with KNO_3_ could significantly ameliorate the effects of waterlogging stress. Supplemental melatonin with KNO_3_ treatment increased chlorophyll content and photosynthetic rate in leaves, increased antioxidant and nitrogen metabolism enzyme activity, and decreased H_2_O_2_ and MDA content in maize seedling leaves under waterlogging stress conditions, as compared to WLM. Melatonin with KNO_3_ also activated the antioxidant system, which effectively scavenged excessive ROS. Melatonin and KNO_3_ also sustained ethylene biosynthesis and production, resulting in a waterlogging stress response. Our results also showed that melatonin with KNO_3_ treatments maintained lower ADH and PDC activities when compared to maize seedlings under waterlogging stress conditions. Overall, the combined effect of melatonin with KNO_3_ was much more effective than the melatonin alone (WLM). Based on our results, we conclude that the application of 100 µM melatonin with 0.50 g KNO_3_ as a seed soaking and foliar application can significantly improve the growth of maize by reducing the detrimental effects of waterlogging stress.

## Figures and Tables

**Table 1:** Physical and chemical composition of the experimental pot soils.

Parameters	Values
Soil organic matter	14.61 g/kg
Available nitrogen	0.88 g/kg
Available phosphorus	48.85 g/kg
Available potassium	96.37 mg/kg
Soil pH	6.83
Water holding capacity	30.31%

**Table 2:** Effect of melatonin with KNO_3_ on dry matter accumulation of root, stem, shoot, leaf, root to shoot ratio and root length of maize seedlings under waterlogging stress conditions.

Methods	Treatments	Root DW (g/plant)	Stem DW (g/plant)	Shoot DW (g/plant)	Leaf DW (g/plant)	Root Shoot Ratio	Root Length (cm/plant)
Foliar Spray	NWL	0.63 ± 0.01 a	0.75 ± 0.02 a	1.70 ± 0.05 a	0.95 ± 0.06 a	0.373	797.59 ± 25.28 a
	WL	0.35 ± 0.03 e	0.48 ± 0.05 b	0.98 ± 0.02 e	0.50 ± 0.03 e	0.361	586.72 ± 24.46 d
	WLM	0.41 ± 0.02 de	0.53 ± 0.03 b	1.09 ± 0.03 d	0.59 ± 0.05 d	0.374	669.73 ± 16.38 c
	WLMN1	0.44 ± 0.01 cd	0.54 ± 0.06 b	1.17 ± 0.04 d	0.63 ± 0.04 d	0.379	730.08 ± 23.98 b
	WLMN2	0.50 ± 0.06 b	0.74 ± 0.05 a	1.58 ± 0.08 b	0.83 ± 0.07 b	0.341	749.55 ± 17.35 b
	WLMN3	0.48 ± 0.05 bc	0.74 ± 0.06 a	1.47 ± 0.04 c	0.73 ± 0.02 c	0.325	744.25 ± 25.04 b
Seed soaking	NWL	0.63 ± 0.07 a	0.97 ± 0.05 a	1.85 ± 0.03 a	0.88 ± 0.02 a	0.413	811.01 ± 18.76 a
	WL	0.35 ± 0.03 d	0.42 ± 0.06 c	1.85 ± 0.03 d	0.49 ± 0.04 d	0.450	562.34 ± 32.51 e
	WLM	0.46 ± 0.03 cd	0.46 ± 0.05 c	1.05 ± 0.08 cd	0.58 ± 0.03 c	0.444	583.20 ± 20.11 de
	WLMN1	0.50 ± 0.05 c	0.49 ± 0.04 c	1.14 ± 0.09 c	0.66 ± 0.06 c	0.439	618.01 ± 21.75 d
	WLMN2	0.69 ± 0.03 ab	0.65 ± 0.10 b	1.46 ± 0.15 b	0.81 ± 0.05 ab	0.479	754.95 ± 27.12 b
	WLMN3	0.61 ± 0.06 b	0.86 ± 0.11 a	1.65 ± 0.16 ab	0.79 ± 0.06 b	0.374	708.47 ± 15.13 c

Control 1 not waterlogging (NWL), Control 2 waterlogging (WL), Control 3 WL + 100 µM Melatonin (WLM), WLM + 0.25 g KNO_3_ (WLMN1), WLM + 0.50 g KNO_3_ (WLMN2), and WLM + 0.75 g KNO_3_ (WLMN3). Data are presented as mean ± SD of three measurements (*n* = 3, biological replicates). Different small letters (a–e) in each column indicate significant differences at *p* ≤ 0.05 (least significant difference (LSD) test).

## Data Availability

Data are available on request due to restrictions, e.g., privacy or ethical. The data presented in this study are available on request from the corresponding author. The data are not publicly available due to the strict management of various data and technical resources within the research teams.

## Abbreviations

KNO_3_	Potassium nitrate
H_2_O_2_	Hydrogen peroxide
MDA	Malondialdehyde
ROS	Reactive oxygen species
SOD	Superoxide dismutase
POD	Peroxidase
CAT	Catalase
APX	Ascorbate peroxidase
N	Nitrogen
NaClO	sodium hypochlorite
NWL	Control not waterlogging
WL	Control waterlogging
WLM	WL + 100 µM Melatonin; WLM + 0.25 g KNO_3_ (WLMN1), WLM + 0.50 g KNO_3_ (WLMN2), WLM + 0.75 g KNO_3_ (WLMN3)
LSD	Least significant difference
DW	Dry weight
GS	Glutamine Synthetase
NR	Nitrate Reductase
GOGAT	Glutamate Synthase
GDH	Glutamate Dehydrogenase
PDC	pyruvate decarboxylase
ADH	Alcohol dehydrogenase
